# Surgical treatment of patients with infective endocarditis: changes in temporal use, patient characteristics, and mortality—a nationwide study

**DOI:** 10.1186/s12872-022-02761-z

**Published:** 2022-07-29

**Authors:** Andreas Dalsgaard Jensen, Lauge Østergaard, Jeppe K. Petersen, Peter Graversen, Jawad H. Butt, Henning Bundgaard, Claus Moser, Morten H. Smerup, Ivy S. Modrau, Kasper Iversen, Niels E. Bruun, Christian Torp-Pedersen, Gunnar Gislason, Andrew Wang, Sigurdur Ragnarsson, Jonas A. Povlsen, Lars Køber, Emil L. Fosbøl

**Affiliations:** 1 Department of Cardiology, The Heart Center, Copenhagen University Hospital Rigshospitalet, Blegdamsvej 9, 2100 Copenhagen, Denmark; 2 Department of Clinical Microbiology, Copenhagen University Hospital Rigshospitalet, Copenhagen, Denmark; 3 Department of Thoracic Surgery, Copenhagen University Hospital Rigshospitalet, Copenhagen, Denmark; 4 Department of Cardiothoracic and Vascular Surgery, Aarhus University Hospital, Aarhus, Denmark; 5 Department of Emergency Medicine, Copenhagen University Hospital Herlev/Gentofte, Herlev, Denmark; 6 Department of Cardiology, Zealand University Hospital, Roskilde, Denmark; 7 Clinical Institutes, Aalborg University, Aalborg, Denmark; 8 Department of Cardiology, Nordsjaellands Hospital, Hilleroed, Denmark; 9 Department of Cardiology, Aalborg University Hospital, Aalborg, Denmark; 10 Department of Cardiology, Copenhagen University Hospital Herlev and Gentofte, Herlev, Denmark; 11 Department of Cardiology, Duke Medical Center, Duke University, Durham, NC USA; 12 Department of Cardiothoracic Surgery, Skaane University Hospital, Lund, Sweden; 13 Department of Cardiology, Aarhus University Hospital, Aarhus, Denmark; 14 The Danish Heart Foundation, Copenhagen, Denmark; 15 Department of Cardiology, Bispebjerg-Frederiksberg Hospital, Copenhagen, Denmark; 16 Department of Clinical Medicine, University of Copenhagen, Copenhagen, Denmark

**Keywords:** Infective endocarditis, Epidemiology, Temporal trend, Cardiac valve surgery

## Abstract

**Background:**

Valve surgery guidelines for infective endocarditis (IE) are unchanged over decades and nationwide data about the use of valve surgery do not exist.

**Methods:**

We included patients with first-time IE (1999–2018) using Danish nationwide registries. Proportions of valve surgery were reported for calendar periods (1999–2003, 2004–2008, 2009–2013, 2014–2018). Comparing calendar periods in multivariable analyses, we computed likelihoods of valve surgery with logistic regression and rates of 30 day postoperative mortality with Cox regression.

**Results:**

We included 8804 patients with first-time IE; 1981 (22.5%) underwent surgery during admission, decreasing by calendar periods (*N* = 360 [24.4%], *N* = 483 [24.0%], *N* = 553 [23.5%], *N* = 585 [19.7%], *P* = < 0.001 for trend). For patients undergoing valve surgery, median age increased from 59.7 to 66.9 years (*P* ≤ 0.001) and the proportion of males increased from 67.8% to 72.6% (*P* = 0.008) from 1999–2003 to 2014–2018. Compared with 1999–2003, associated likelihoods of valve surgery were: Odds ratio (OR) = 1.14 (95% CI: 0.96–1.35), OR = 1.20 (95% CI: 1.02–1.42), and OR = 1.10 (95% CI: 0.93–1.29) in 2004–2008, 2009–2013, and 2014–2018, respectively. 30 day postoperative mortalities were: 12.7%, 12.8%, 6.9%, and 9.7% by calendar periods. Compared with 1999–2003, associated mortality rates were: Hazard ratio (HR) = 0.96 (95% CI: 0.65–1.41), HR = 0.43 (95% CI: 0.28–0.67), and HR = 0.55 (95% CI 0.37–0.83) in 2004–2008, 2009–2013, and 2014–2018, respectively.

**Conclusions:**

On a nationwide scale, 22.5% of patients with IE underwent valve surgery. Patient characteristics changed considerably and use of valve surgery decreased over time. The adjusted likelihood of valve surgery was similar between calendar periods with a trend towards an increase while rates of 30 day postoperative mortality decreased.

**Supplementary Information:**

The online version contains supplementary material available at 10.1186/s12872-022-02761-z.

## Introduction

Infective endocarditis (IE) is associated with significant morbidity and high in-hospital mortality rates between 14 and 24% [1–4]. IE status or disease progression may necessitate valve surgery, and European and American guidelines have comparable recommendations for surgery, which have been similar over decades [5–7]. The incidence rate (IR) of IE has increased over the past three decades; more than doubling in Denmark over the last 20 years [1, 2, 4, 8–12]. IE patient characteristics have also changed markedly across the last decades with a higher median age and an increasing burden of comorbidities although previous disposing factors like rheumatic fever have decreased substantially [1, 9, 12, 13]. In parallel to the increase in the incidence of IE, an increase in the use of cardiac surgery in IE was anticipated almost 20 years ago [14]. Previous studies report significant variations in the proportion of patients with IE undergoing cardiac surgery (10–48%) [1, 3, 13, 15–17]. In a recent study by Habib et al. 51% of the patients with IE underwent cardiac surgery with data from voluntarily participating tertiary treatment centres (European Society of Cardiology EURObservational Research Programme (ESC-EORP) European Endocarditis (EURO-ENDO)), potentially affected by selection bias [18]. A previous study by Kanafani et al. found that 73% of patients with IE underwent cardiac surgery using data from the International Collaboration on Endocarditis Prospective Cohort Study (ICE-PCS) which is a cohort of tertiary treatment centres with an inherent referral bias [19]. Accordingly, guidelines on surgery in IE may reflect recommendations based on data where proportions of patients who undergo valve surgery are not representative for the complete cohort of patients with IE. Data on the use of valve surgery in IE are warranted from unselected nationwide cohorts to ensure continuous monitoring of the disease and to elucidate nationwide practice patterns. We aimed to examine temporal trends in valve surgery among patients with IE, and report patient characteristics and outcomes using nationwide registries in Denmark from 1999 to 2018, thus addressing the need for contemporary epidemiological tracking of valve surgery in IE.

## Methods

This nationwide and population-based cohort study was conducted for a 20 year period from January 1st, 1999 until December 31st, 2018.

### Data sources

In Denmark, linking health care registries on a nationwide scale is possible due to the unique personal identifier (10-digits) given at birth or when residing in Denmark for more than three months. Researchers are able to consider the entire population of Denmark as a cohort for epidemiological research [20].

The Danish Civil Registration System holds electronic on date of birth, migration status, and sex [20]. The Danish National Patient Registry holds electronic records of every hospitalization of every citizen since 1977 based solely on the treating physicians’ discharge summary, including primary (mandatory) and secondary diagnosis codes according to the International Classification of Diseases (ICD) [21]. ICD-8 was used before 1994, ICD-10 after 1994 and the Nordic-Medico-Statistical Committee’s (NOMESCO’s) classification of surgical procedures was added in 1996, while implantation of cardiac implantable electronic devices (CIED) and dialysis were added in 2000 [21]. The Danish Registry of Causes of Death holds electronic records of date of death [22]. The Danish National Prescription Registry holds electronic records of dispensed prescription drugs since 1994 including date of dispense and package size (organized by the Anatomical Therapeutic Chemical Classification System [ATC]) [23]. This manuscript fully conforms to national Danish law regarding ethics in registry-based studies.

### Study population, follow-up, and outcome

**Fig. 1:**
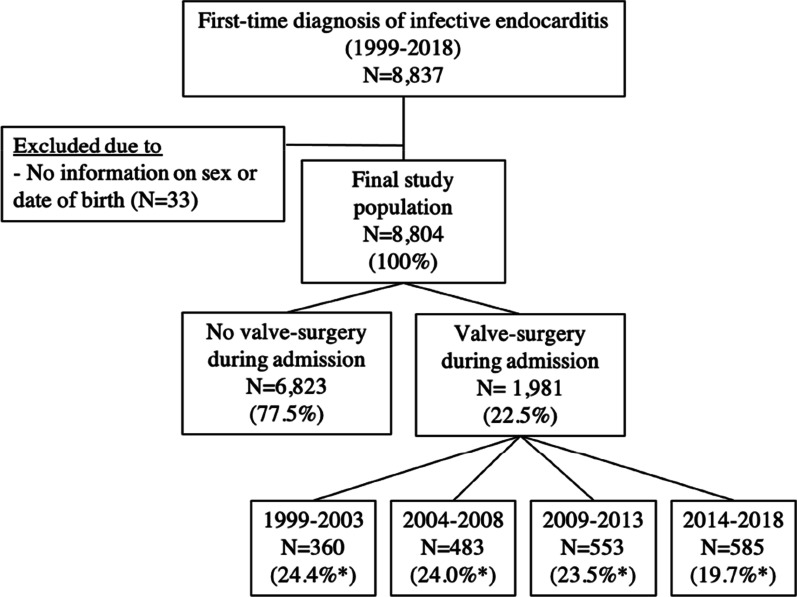
Flowchart of patient inclusion. Percentage marked with “*” is calculated as the number of patients with valve surgery per calendar period relative to the total number of patients per calendar period

Using Danish registries, we identified patients with IE (ICD-10: I33.x, I38.x, I39.8, ICD-8: 421) and included their first admission with IE to a hospital in Denmark (1999–2018) with no missing information on sex or date of birth, Fig. 1. We used the same criteria for the inclusion of IE patients as Ostergaard et al. who found a positive predictive value (PPV) of 90% for the IE diagnosis using the Danish National Patient Registry [24]. We accounted for transfers between hospital departments by tracking entries in The Danish National Patient Registry related to the study population. If there was less than 24 h between discharge and admission the patient was considered as transferred between departments under the same admission. The study population was grouped into the 5 year time spans of calendar periods: 1999–2003, 2004–2008, 2009–2013, and 2014–2018 for the purpose of analysis. For the outcome of postoperative mortality, patients were followed from date of valve surgery until 30 days after surgery, emigration, death, or December 31st, 2018 (whichever came first).

### Covariates

We derived comorbidities and concomitant pharmacotherapy for every patient in our study population, Additional file 1: Table S1 (specific ICD-10, ICD-8 and ATC codes used). Comorbidities were defined based on diagnoses given prior to admission in conjunction with an in- or outpatient hospital contact. Pharmacotherapy prior to admission was defined as every redeemed prescription within six months prior to admission. Hypertension was derived as use of two or more antihypertensive drugs within six months prior to admission as done previously [25]. We identified patients with a history of abuse of opioids, sedative/hypnotics, cocaine derived by ICD-10 codes, Additional file 1: Table S1. From the registries used, we were not able to characterize if a patient with substance abuse was using an intravenous drug type.

ICD-10 and ICD-8 diagnosis codes contains no specific information on location of IE (right vs. left, native vs. prosthetic, device related) why we estimated the presumed number of patients with isolated RS-IE. In Denmark, left-sided IE is treated by cardiologist and/or thoracic surgeons. Patients with isolated native right-sided valve involvement (not CIED infection and not involvement of left-sided valves) without indication of valve-surgery are treated at departments of infectious diseases. We defined presumed isolated right-sided IE (RS-IE) as a patient with IE who either underwent an extraction of their CIED or were admitted to an infection disease ward. Patients with any type of valve-surgery were presumed as having left-sided IE. Only patients having an extraction of a previously implanted CIED or patients admitted more than half of their admission at an infectious disease ward (with a total admission-time less than three weeks) was counted as presumed isolated RS-IE.

### Statistics

Baseline characteristics were presented by calendar periods (1999–2003, 2004–2008, 2009–2013, and 2014–2018). Categorical variables were presented as counts (N) and proportions (in %). Continuous variables were presented as medians and 25–75th percentile. Differences in baseline characteristics between calendar periods were tested by Cochrane-Armitage test for trend (categorical covariates) and Kruskal–Wallis test (continuous covariates).

Differences in the proportion of patients undergoing valve surgery during admission were tested for trend for calendar time: overall and per age-group (< 40 years, 40–64 years, 65–75 years, and ≥ 76 years) using the Cochrane-Armitage test for trend.

To calculate the IR of IE per 100,000 person-years (PY) by calendar period, each person in Denmark was followed from whichever came last of January 1st (of the respective calendar year), immigration date, or birth date until whichever came first of admission for IE, death, emigration, or December 31st (in the respective calendar year). IR and 95% confidence-intervals (95% CI) were calculated assuming a Poisson distribution.

We used multivariable logistic regression to compute the odds ratios (OR) of undergoing valve surgery during IE admission for the study periods. Age was tested for linearity and was not met in the logistic regression model and age was categorized accordingly (< 40 years, 40–49 years, 50–59 years, 60–69 years, 70–79 years, and ≥ 80 years) The following covariates were included in the logistic regression model: calendar period, age groups (< 40, 40–49, 50–59, 60–69, 70–79, ≥ 80 years), sex, prior prosthetic heart valve, diabetes, renal disease, liver disease, atrial fibrillation/flutter (AF), congestive heart failure, myocardial infarction, disease of the mitral valve, disease of the aortic valve, ischemic/haemorrhagic stroke, chronic obstructive lung disease, and malignancy. In a subgroup analysis, the population was limited to the calendar years 2000–2018, as data on CIED and dialysis was only available from 2000. In additional analyses we examined whether selected covariates (prior prosthetic heart valve, sex, age group, renal disease, liver disease, congestive heart failure. and stroke) were modified by calendar period on the outcome of undergoing valve surgery.

For patients undergoing valve surgery, 30 day postoperative mortality (all-cause) per calendar period was estimated using Kaplan–Meier estimates. Log-rank testing was used to assess differences between calendar periods. For patients undergoing surgery, hazard ratios (HR) of 30 day postoperative mortality (all-cause) were estimated using multivariable Cox proportional hazard regression modelling. We used the same covariates in this model as in the previous specified multivariable model. An additional analysis was computed using same covariates as previously mentioned with addition of CIED and dialysis (study period 2000–2018).

Continues variables were tested for linearity and reported if violated. Interaction between calendar period and sex, age group, and prior prosthetic heart valve was tested and reported if violated. The proportional hazards assumption was assessed in graphical terms using Martingale’s residuals and reported if violated.

The level of statistical significance was *P* ≤ 0.05. Data management and statistical analysis were conducted using SAS software 9.4 (SAS Institute, Inc., Cary, NC, USA) and the statistical software R version 3.5.0 [26].

## Results

Overall, 8804 patients were admitted with first-time IE between 1999 and 2018, Fig. 1. The distribution by calendar periods was: *N* = 1475 (16.7%), *N* = 2011 (22.8%), *N* = 2355 (26.8%), and *N* = 2963 (33.7%) in 1999–2003, 2004–2008, 2009–2013, and 2014–2018, respectively. The IR of IE increased over calendar periods: IR = 5.2 (95% CI: 5.0–5.5) in 1999–2003, IR = 7.1 (95% CI: 6.8–7.4) in 2004–2008, IR = 8.2 (95% CI: 7.9–8.6) in 2009–2013, and IR = 10.2 (95% CI: 9.9–10.6) in 2014–2018 (per 100,000 PY).

Out of 8804 patients with IE, 1981 (22.5%) patients underwent valve surgery during the admission, Fig. 1. By calendar period, this corresponded to: *N* = 360 (24.4%) in 1999–2003, *N* = 483 (24.0%) in 2004–2008, *N* = 553 (23.5%) in 2009–2013, and *N* = 585 (19.7%) in 2014–2018 (*P* ≤ 0.001 for decreasing trend).

### Patient characteristics

Patients undergoing valve surgery were younger than patients not undergoing valve surgery, though the median age increased for both patient groups between 1999 and 2018, Table 1. The proportion of males was higher for patients undergoing valve surgery and the proportion of male patients increased over calendar time, Table 1. When comparing study periods, patients undergoing valve surgery were more often characterized by having diabetes, renal disease, CIEDs, AF, ischemic heart disease, disease of the aortic valve, ischemic/haemorrhagic stroke, and malignancy in the later study periods, Table 1. Patients not undergoing valve surgery had a higher burden of comorbidities compared with patients undergoing valve surgery, Table 1. The proportion of presumed isolated RS-IE increased from 3.0% in 1999–2003 to 8.7% in 2014–2018, Table 1.

**Table 1:** Baseline characteristics

	First-time diagnosis of infective endocarditis (IE) between 1999 and 2018										
	Overall (100%)	Valve surgery during admission *N* = 1981 (22.5%)					No valve surgery during admission *N* = 6823 (77.5%)				
	1999–2018 *N* = 8804 (100%)	1999–2003 *N* = 360 (24.4%*)	2004–2008 *N* = 483 (24.0%*)	2009–2013 *N* = 553 (23.5%*)	2014–2018 *N* = 585 (19.7%*)	*P* value	1999–2003 *N* = 1115 (75.6%*)	2004–2008 *N* = 1528 (76.0%*)	2009–2013 *N* = 1802 (76.5%*)	2014–2018 *N* = 2378 (80.3%*)	*P* value
*Age group*											
< 40 years	596 (6.8%)	48 (13.3%)	42 (8.7%)	49 (8.9%)	48 (8.2%)	0.024	124 (11.1%)	109 (7.1%)	92 (5.1%)	84 (3.5%)	< 0.001
40–49 years	613 (7.0%)	47 (13.1%)	57 (11.8%)	69 (12.5%)	49 (8.4%)	0.031	93 (8.3%)	106 (6.9%)	103 (5.7%)	89 (3.7%)	< 0.001
50–59 years	1107 (12.6%)	90 (25.0%)	113 (23.4%)	109 (19.7%)	97 (16.6%)	< 0.001	133 (11.9%)	161 (10.5%)	183 (10.2%)	221 (9.3%)	0.017
60–69 years	1893 (21.5)	90 (25.0%)	152 (31.5%)	169 (30.6%)	165 (28.2%)	0.561	200 (17.9%)	293 (19.2%)	393 (21.8%)	431 (18.1%)	0.902
70–79 years	2627 (29.8)	76 (21.1%)	103 (21.3%)	133 (24.1%)	199 (34.0%)	< 0.001	342 (30.7%)	450 (29.5%)	515 (28.6%)	809 (34.0%)	0.013
> 79 years	1968 (22.4%)	9 (2.5%)	16 (3.3%)	24 (4.3%)	27 (4.6%)	0.071	223 (20.0%)	409 (26.8%)	516 (28.6%)	744 (31.3%)	< 0.001
Age (median, 25–75th percentile)	70.8 (59.2–79.1)	59.7 (49.6–69.1)	62.2 (52.3–69.8)	63.1 (52.3–71.6)	66.9 (55.2- 73.0)	< 0.001	70.2 (55.2, 78.3)	72.4 (60.2, 80.5)	72.6 (63.0, 81.2)	74.8 (65.8, 81.7)	< 0.001
Male	5762 (65.4%)	244 (67.8%)	352 (72.9%)	431 (77.9%)	425 (72.6%)	0.008	640 (57.4%)	935 (61.2%)	1208 (67.0%)	1525 (64.1%)	< 0.001
Length of hospital admission (days, median, 25–75th percentile)	42.0 (29.0, 52.0)	55.0 (41.0–72.0)	49.0 (38.0–66.0)	48.0 (40.0–64.0)	45 .0 (35.0–56.0)	< 0.001	39.0 (27.0–51.0)	41.0 (26.0–52.0)	39.0 (26.0–50.0)	35.0 (25.0–47.0)	< 0.001
Days from admission until surgery (days, median, 25–75th percentile)	–	18.5 (8.0–34.0)	13.0 (5.0–25.0)	9.0 (4.0–20.0)	6.0 (3.0–13.0)	< 0.001	–	–	–	–	–
Extraction of a previously implanted CIED and valve surgery	21 (0.2%)	< 4 (< 1.1%)	< 4 (< 0.8%)	6 (1.1%)	11 (1.9%)	0.011	–	–	–	–	–
Presumed isolated RS-IE^1^	466 (5.3%)	–	–	–	–	–	33 (3.0%)	68 (4.5%)	157 (8.7%)	208 (8.7%)	< 0.001
Of them, extraction of a previously implanted CIED and no valve surgery	367 (78.8%)	–	–	–	–	–	4 (12.1%)	50 (7.4%)	133 (84.7%)	180 (86.5%)	< 0.001
Of them, more than half of admission at an infectious disease ward and no valve surgery	99 (21.4%)	–	–	–	–	–	29 (87.9%)	18 (26.5%)	24 (15.3%)	28 (13.5%)	0.314
*Comorbidity, medical history of:*											
Prosthetic heart valve (prior to admission)	1450 (16.5%)	26 (7.2%)	50 (10.4%)	93 (16.8%)	117 (20.0%)	< 0.001	78 (7.0%)	212 (13.9%)	346 (19.2%)	528 (22.2%)	< 0.001
Of them, number, and proportion of TAVI	176 (12.1%)	0 (0.0%)	0 (0.0%)	< 4 (< 4.3%)	5 (4.3%	0.012	0 (0.0%)	0 (0.0%)	33 (9.5%)	136 (25.8%)	< 0.001
Diabetes	1739 (19.8%)	34 (9.4%)	47 (9.7%)	85 (15.4%)	105 (17.9%)	< 0.001	145 (13.0%)	258 (16.9%)	406 (22.5%)	659 (27.7%)	< 0.001
Renal disease	1159 (13.2%)	20 (5.6%)	31 (6.4%)	50 (9.0%)	53 (9.1%)	0.020	84 (7.5%)	178 (11.6%)	300 (16.6%)	443 (18.6%)	< 0.001
Dialysis^2^	495 (5.6%)	7 (1.9%)	14 (2.9%)	26 (4.7%)	24 (4.1%)	0.042	28 (2.5%)	93 (6.1%)	134 (7.4%)	169 (7.1%)	< 0.001
Liver disease	622 (7.1%)	20 (5.6%)	26 (5.4%)	22 (4.0%)	30 (5.1%)	0.603	70 (6.3%)	108 (7.1%)	141 (7.8%)	205 (8.6%)	0.009
CIED^2^	1066 (12.2%)	4 (1.1%)	15 (3.1%)	26 (4.7%)	36 (6.2%)	< 0.001	24 (2.2%)	134 (8.7%)	321 (17.8%)	507 (21.3%)	< 0.001
Atrial fibrillation/flutter	2105 (23.9%)	40 (11.1%)	55 (11.4%)	80 (14.5%)	99 (16.9%)	0.003	184 (16.5%)	328 (21.5%)	515 (28.6%)	804 (33.8%)	< 0.001
Congestive heart failure	1946 (22.1%)	41 (11.4%)	66 (13.7%)	80 (14.5%)	66 (11.3%)	0.848	223 (20.0%)	345 (22.6%)	486 (27.0%)	639 (26.9%)	< 0.001
Myocardial infarction	1014 (11.5%)	14 (3.9%)	26 (5.4%)	36 (6.5%)	38 (6.5%)	0.081	106 (9.5%)	197 (12.9%)	265 (14.7%)	332 (14.0%)	< 0.001
Disease of the mitral valve	783 (8.9%)	46 (12.8%)	43 (8.9%)	50 (9.0%)	44 (7.5%)	0.015	110 (9.9%)	134 (8.8%)	146 (8.1%)	210 (8.8%)	0.374
Disease of the aortic valve	2296 (26.1%)	63 (17.5%)	112 (23.2%)	155 (28.0%)	195 (33.3%)	< 0.001	191 (17.1%)	314 (20.5%)	510 (28.3%)	756 (31.8%)	< 0.001
Ischemic/haemorrhagic stroke	1061 (12.0%)	14 (3.9%)	33 (6.8%)	45 (8.1%)	52 (8.9%)	0.004	103 (9.2%)	180 (11.8%)	265 (14.7%)	369 (15.5%)	< 0.001
Chronic obstructive lung disease	1054 (12.0%)	18 (5.0%)	32 (6.6%)	43 (7.8%)	45 (7.7%)	0.102	130 (11.7%)	192 (12.6%)	266 (14.8%)	328 (13.8%)	0.046
Malignancy	1623 (18.4%)	37 (10.3%)	45 (9.3%)	57 (10.3%)	95 (16.2%)	0.002	164 (14.7%)	270 (17.7%)	366 (20.3%)	589 (24.8%)	< 0.001
Drug abuse	147 (1.7%)	< 4 (< 1.1%)	< 4 (< 0.8%)	4 (0.7%)	< 4 (< 0.7%)	0.413	22 (2.0%)	27 (1.8%)	22 (1.2%)	26 (1.1%)	0.017
*Pharmacotherapy within 6 months prior to hospital admission*											
Hypertension	4261 (48.4%)	93 (25.8%)	179 (37.1%)	233 (42.1%)	241 (41.2%)	< 0.001	409 (36.7%)	750 (49.1%)	1027 (57.0%)	1329 (55.9%)	< 0.001
Glucose lowering medication	1351 (15.3%)	28 (7.8%)	37 (7.7%)	70 (12.7%)	82 (14.0%)	< 0.001	113 (10.1%)	199 (13.0%)	318 (17.6%)	504 (21.2%)	< 0.001
Lipid lowering medication	2674 (30.4%)	26 (7.2%)	104 (21.5%)	164 (29.7%)	195 (33.3%)	< 0.001	82 (7.4%)	387 (25.3%)	711 (39.5%)	1005 (42.3%)	< 0.001
Oral anticoagulants	2137 (24.3%)	48 (13.3%)	74 (15.3%)	105 (18.9%)	133 (22.7%)	< 0.001	178 (15.9%)	323 (21.1%)	458 (25.4%)	818 (34.4%)	< 0.001

This table shows the associated covariates (comorbidities and pharmacotherapy) for patients with infective endocarditis per calendar period (1999–2003, 2004–2008, 2009–2013, 2014–2018) subdivided by valve surgery during admission

*CIED*, cardiac implantable electronic device, *RS-IE*, right-sided infective endocarditis

Percentage marked with “*” is calculated as the number of patients with/without valve surgery per calendar period relative to the total number of patients per calendar period

^1^Patients were presumed having right sided IE if more than half of their current admission was at an infectious disease ward or if they had a prior CIED and subsequently underwent an extraction during admission

^2^Data on dialysis and CIED was available from ≥ 2000

### Temporal changes in the proportion of patients undergoing valve surgery

**Fig. 2:**
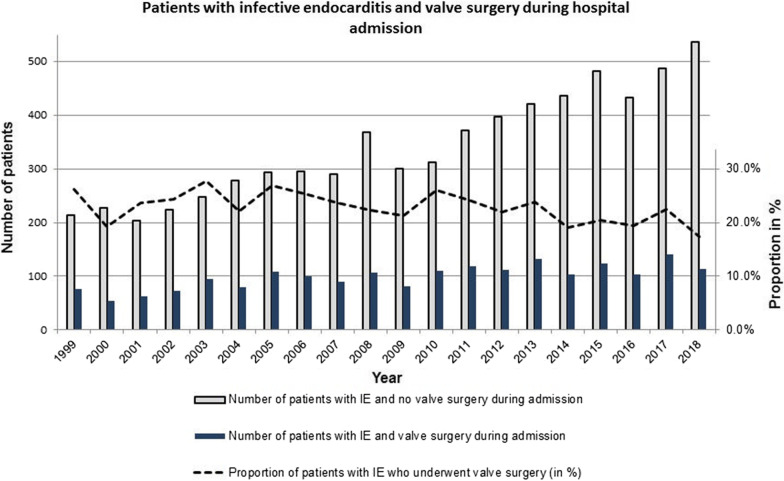
Number of IE-patients and valve surgery during admission. The figure shows the total number of patients with first-time IE with/without surgery during admission. Furthermore, the figure shows the proportion of patients with IE who undergo surgery during admission (in %) as well as the postoperative mortality (30 days, Kaplan–Meier estimates, in %)

**Fig. 3:**
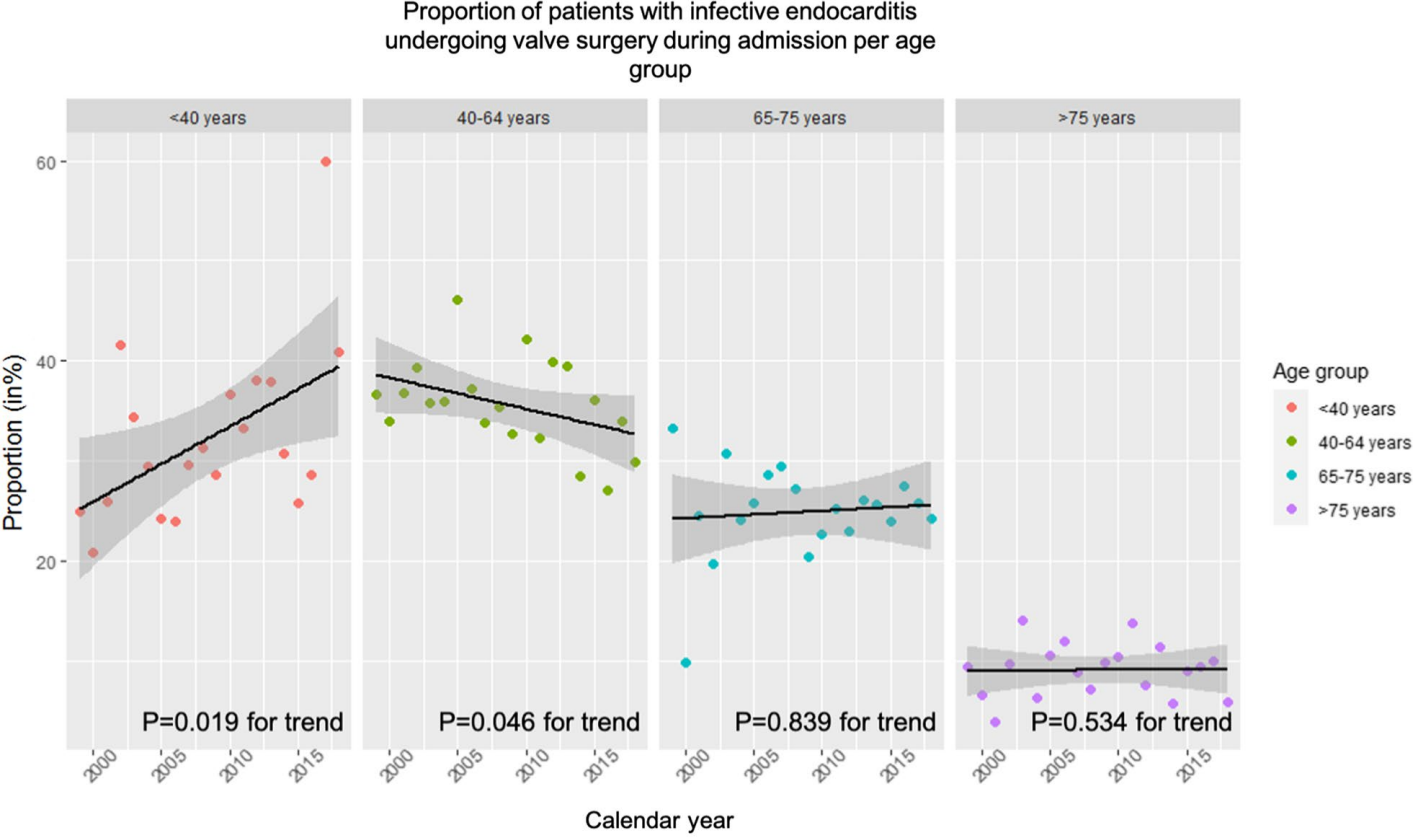
The figure shows the proportion of patients with IE who underwent surgery during their admission by calendar-year by the age groups < 40 years, 40–64 years, 65–75 years, > 75 years. IE: Infective endocarditis

Numerically, the number of IE patients who underwent valve surgery increased, yet the proportion of IE-patients undergoing valve surgery decreased between 1999 and 2018 (*P* ≤ 0.001 for trend), Fig. 2. The proportion of patients treated with valve surgery increased from 25.0% in 1999 to 40.9% in 2018 for the patients aged < 40 years (*P* = 0.019 for trend). For patients aged 40–64 years, the proportion decreased from 36.7 to 29.9% in the same period (*P* = 0.046 for trend), Fig. 3. No significant changes were found for the age groups 65–75 years or > 75 years, Fig. 3.

### Temporal changes in choice of surgical modality

Of the 1981 patients who underwent valve surgery between 1999 and 2018, 1788 (90.3%) patients underwent a valve replacement, and 193 (9.7%) patients underwent valve-repair, Additional file 1: Table S1. The proportion of valve-replacements relative to valve-repairs remained stable between 1999 and 2018 while an increase in the use of biological prostheses accompanied by a decrease in the use of mechanical prostheses were identified, Additional file 1: Table S2.

### Adjusted likelihood of undergoing valve surgery and factors associated with valve surgery

In the adjusted analyses and relative to 1999–2003, the associated odds of valve surgery were significantly higher in 2009–2013 while no statistically significant differences were found for 2003–2004, and 2014–2018, Table 2.

**Table 2:** Odds ratio of valve surgery during admission and hazard ratio of 30 day postoperative mortality for patients with infective endocarditis in the study period 1999–2018

	Valve surgery during admission		30 day postoperative mortality	
Calendar periods	Odds ratio (95% CI) Adjusted^1^	*P*-value	Hazard ratio (95% CI) Adjusted^2^	*P*-value
1999–2003	1.00 (ref.)	–	1.00 (ref.)	–
2004–2008	1.14 (0.96–1.35)	0.133	0.96 (0.65–1.41)	0.840
2009–2013	1.20 (1.02–1.42)	0.030	0.43 (0.28–0.67)	< 0.001
2014–2018	1.10 (0.93–1.29)	0.266	0.55 (0.37–0.83)	0.004

This table shows the associated odds ratios of valve surgery and 30 day postoperative mortality when adjusting for covariates for patients with first-time infective endocarditis between 1999 and 2018

Odds Ratio > 1 = increased likelihood. Hazard ratio > 1 = increased rate. Reference (ref.)

^1^Model adjusted for: calendar period, age groups, sex, prior prosthetic heart valve, diabetes, renal disease, liver disease, atrial fibrillation/flutter, congestive heart failure, myocardial infarction, disease of the mitral valve, disease of the aortic valve, ischemic/haemorrhagic stroke, chronic obstructive lung disease, malignancy. ^2^Model adjusted for: calendar period, age (continues), sex, prior prosthetic heart valve, diabetes, renal disease, liver disease, atrial fibrillation/flutter, congestive heart failure, myocardial infarction, disease of the mitral valve, disease of the aortic valve, ischemic/haemorrhagic stroke, chronic obstructive lung disease, malignancy

Regarding the prespecified covariates in the adjusted analysis, we identified an increase in the associated ORs of valve surgery during admission for male sex and the age groups 40–49 years and 50–59 as compared with < 40 years, Additional file 1: Table S3. For the prespecified covariates, we found a decrease in the associated ORs of valve surgery during admission for prior prosthetic heart valve, renal disease, liver disease, stroke, congestive heart failure, and the age groups 70–79 years and > 79 years as compared with < 40 years, Additional file 1: Table S3. No difference across calendar periods were identified for the prespecified covariates and the associated chance of undergoing valve surgery, Additional file 1: Table S3.

### Postoperative mortality

The crude 30 day postoperative mortality for patients with IE were 12.7% (95% CI: 9.6–16.5%), 12.8% (95% CI: 10.0–16.0%), 6.9% (95% CI: 5.0–9.2%), and 9.7% (95% CI: 7.5–12.2%) for the calendar periods 1999–2003, 2004–2008, 2009–2013, and 2014–2018, respectively (*P* = 0.005 for difference). Over time and in adjusted analyses, there was a decrease in the associated HR for 30 day postoperative mortality for the calendar periods 2009–2013 and 2014–2018, while the HR remained insignificantly different in the calendar period 2004–2008 relative to 1999–2003, Table 2.

### Subgroup analyses: including CIED and dialysis in the study period 2000–2018

In the adjusted model including CIED and dialysis, there was an increase in the associated OR of valve surgery for the calendar periods 2009–2013 and 2014–2018, while the associated ORs were without statistically significant differences in 2004–2008 relative to 2000–2003, Additional file 1: Table S3. In the adjusted analysis including CIED and dialysis, the associated HR of 30 day postoperative mortality was without statistically significant difference in 2004–2008, while the associated HRs was significantly lower in 2009–2013 and 2014–2018 relative to 1999–2003, Additional file 1: Table S4.

## Discussion

The present study explored Danish nationwide temporal trends in the use of valve surgery among patients with IE. The present study had three major findings. First, the characteristics of IE patients who underwent valve surgery changed during the study period (1999–2018), that is: increase in the median age, increase in the male predominance, and increase in the burden of comorbidities (including increases in the proportion of patients with diabetes, renal disease, prior prosthetic heart valves and CIEDs). Second, crude number of patients with valve surgery increased while the crude proportion of valve surgery among patients with IE decreased over time. However, when patient characteristics were considered, the associated likelihood of valve surgery was similar between calendar periods with a trend towards an increase in the use of valve surgery. Third, 30 day postoperative mortality in IE patients decreased between 1999 and 2018. In this nationwide study covering all patient with IE independent of treating health care level one in four patients with IE underwent valve surgery. The generally reported proportion of patients with IE undergoing valve surgery is more around 40–50% [5, 16, 18] where this study reports a proportion less than half that. The discrepancy is most likely related to the issue that the majority of reports on this topic stems from tertiary referral centres. Thus, taken as a whole, our data suggest that prior studies using data from tertiary centres significantly overestimate the use of valve surgery in IE.

In a study by Olmos et al*.*, referring to guidelines on the treatment on IE, it was estimated that cardiac surgery is required in more than 50% of patients with IE - higher among patients with left-sided IE [16]. This estimate was confirmed in a recent study by Habib et al*.*, using data from the European Endocarditis Registry cohort of tertiary treatment centres (EURO-ENDO) [18]. Although surgery might be indicated, it is assumed that contraindications for surgery have also increased over time and patients with IE are generally older, more fragile and more co-morbid in recent years [1, 13]. We found that valve surgery was used significantly less in patients with first-time IE (19.8% in 2014–2018) in the present study as compared to the studies by Habib et al. and Olmos et al*.* In general, the reported proportions of valve surgery among IE-patients, are higher in studies from tertiary treatment centres than nationwide studies [1, 3, 13, 19] which corresponds to relatively low use of valve surgery in the present study. In a Spanish nationwide study by Olmos et al*.* using data from the Spanish National Health Service, the overall proportion of cardiac surgery among patients with IE diagnoses at a regional hospital was 26.5% in 2014 [1]. The proportion of surgery in the study by Olmos et al*.* is comparable to the proportion in the present study, however, Olmos et al. found an increasing trend between 2003 and 2014 while this study found a decreasing trend. Other nationwide studies have shown an increase in the proportion of patients undergoing valve surgery for IE [11, 27]. Possible explanations could be that utilization of modern diagnostic tools and surgical treatment is applied differently in different countries, which could be a subject of interest in the worldwide clinical practice of IE.

The difference between proportions of valve surgery among patients diagnosed and managed at regional hospitals and those diagnosed or referred to tertiary centers is likely explained by referral-bias. It might be that the elderly and those with largest burden of comorbidities and highest perioperative risk or those with relatively mild disease are not being transferred to tertiary centres for closer examination with the purpose of surgery.

We found a relatively low utilization of valve surgery in the present study, and a decrease in the 30 day postoperative mortality. We were not able to assess direct explanations for this decrease in mortality. Some studies have suggested better outcomes for patients with IE due to improved critical care and surgical techniques [28, 29]. Further, diagnostics modalities such as echocardiography and positron emission tomography have improved in recent years [30–32] which may lead to patients being diagnosed earlier and more patients being diagnosed than previously. Some of the antibiotic treatment regimens for IE have also changed during the study period [33] which might have improved outcomes for patients. In addition, there has been a centralizing of surgical expertise in high-volume centres in Denmark. In all, this could explain why mortality has decreased for patients undergoing surgery.

Other studies have evaluated mortality in patients with IE undergoing valve surgery, however, often in specific sub-groups (aortic/mitral-valve only, right-sided only, native- vs. prosthetic-valve-IE etc.) with in-hospital mortality ranging from 11 to 38% [15, 34, 35]. In a study on IE patients treated conservatively compared to patients treated surgically in 1998–2014, Cresti et al*.* found an in-hospital mortality of 22.8% and a 1 year mortality of 30.4% among surgically treated, however, this study was limited by restricted power (*N* = 170) [2]. In comparison, the present study shows a 30 day postoperative mortality which decreased from 12.7% (95% CI:9.6–16.5) to 9.7% (95% CI 7.5–12.2%) in 1999–2003 and 2014–2018, respectively.

This study delivers novel data on outcome for IE patients undergoing valve surgery, thus addressing the need for contemporary, nationwide data on patients with IE. ICD-10 codes do not allow for differentiation of the localization of IE (right-sided, left-sided, or related to CIED). However, we calculated the number of presumed isolated RS-IE which constituted 5.5% of the total study population. Another Danish study by Lassen et al*.*, examined patients with IE in the Southern Region of Denmark (approximately 20% of the Danish population) in the period from 2007 to 2017 [36]. Lassen et al*.* found that 11% of patients had RS-IE related to a CIED, 6% had RS-IE without relation to a CIED, and 83% had left-sided IE [36]. Lassen et al. did not examine temporal trends in the proportion of RS-IE. If the proportion of RS-IE is increasing it could partially explain the decreasing proportion of valve surgery observed in the present study since RS-IE less often requires valve surgery. In the present study, the number of presumed isolated RS-IE increased from 3.0 to 8.7% for patients not undergoing valve surgery. However, this increment in patients with IE and CIED extraction must be interpreted cautiously as data before the year 2000 was not available. In addition, efforts to assess the number of people who inject drugs (PWIDs) by the Danish Health Authorities suggest a decrease in the number and proportion of PWIDs in Denmark over the last 20 years [37, 38] and this may in part explain our findings and is also similar to the decreasing proportion of PWIDs found in the Spanish nationwide study Olmos et al. [1].

Though speculative, results on presumed isolated RS-IE from the present study and the number of RS-IE in the study by Lassen et al*.* suggests that RS-IE remains a small proportion of patients with IE in Denmark. It is not possible to conclude if the observed decrease in the proportion of patients with IE undergoing valve surgery is driven by a sharp increase in RS-IE, however, results suggests that this is not the case in Denmark.

### Strengths and limitations

The present study delivers nationwide, unselected data on the use of valve surgery among patients with IE in the period 1999–2018. The PPV of IE, when using the validated criteria by Ostergaard et al. is 90% and Danish registries are of high quality, [20–24] improving the reliability of our results. The nationwide and population-based design of the present study minimizes the risk of referral bias and patients included were followed for up to 20 years. The present study has several limitations. First, clinical data on microbial aetiology, antimicrobial therapy, echocardiography, location of infection (i.e. valve involvement), prosthetic valve involvement, relation to a CIED was not available. Second, history of intravenous drug abuse was not available and could have added to the knowledge of patient outcomes in specific subgroups. Third, the selection of patients for valve surgery might be affected by relative contraindications such as known comorbidities or high age. In the present study, data necessary for the calculation of operative risk from registries was not possible due to missing key variables. Previous multinational results have shown that approximately 1 in 4 left-sided IE cases with an indication for surgery do not undergo surgery because of operative risk [34]. Fourth, no information on the specificity and sensitivity of the IE-diagnosis in The Danish National Patient Registry is available and the study on the PPV of the IE diagnosis by Ostergaard et al. was performed on data in the period 2010–2012 and differences in the PPV of the diagnosis may exist.

## Conclusions

In this nationwide study of patients with IE between 1999 and 2018, we found that characteristics of IE patients who underwent valve surgery changed markedly; age and comorbidity increased over time. When accounting for patient characteristics the associated likelihood of valve surgery was similar between calendar periods with a trend towards an increase and the associated 30 day postoperative mortality decreased. Our study suggests that surgical treatment in IE is used less than reported in previous literature.

## Supplementary Information

**Additional file 1**: **Table S1**. Overall ICD-, Procedure- and ATC-codes. **Table S2**. Type of valve-procedure during admission. **Table S3**. Odds ratio of valve surgery during admission for prespecified covariates in the study period 1999–2018 overall and when stratifying by calendar periods. **Table S4**. Odds ratio of valve surgery during admission and hazard ratio of 30-day postoperative mortality for patients with infective endocarditis in the study period 2000-2018 including CIED and dialysis.

## Data Availability

Due to rules of anonymity, Danish law prohibits any publication of raw data from Statistics Denmark.
