# Supplementary material for: Surgical treatment of patients with infective endocarditis: changes in temporal use, patient characteristics, and mortality—a nationwide study
Source: BMC Cardiovasc Disord. 2022 Jul 29;22:338. doi: 10.1186/s12872-022-02761-z (PMC9336053; doi:10.1186/s12872-022-02761-z)
Supplement: Supplementary file 1 — Additional file 1: Table S1. Overall ICD-, Procedure- and ATC-codes. Table S2. Type of valve-procedure during admission. Table S3. Odds ratio of valve surgery during admission for prespecified covariates in the study period 1999–2018 overall and when stratifying by calendar periods. Table S4. Odds ratio of valve surgery during admission and hazard ratio of 30-day postoperative mortality for patients with infective endocarditis in the study period 2000-2018 including CIED and dialysis. [file 12872_2022_2761_MOESM1_ESM.docx]

**Supplementary Material**

**Supplementary Table 1**

# *Supplementary Table 1: Overall ICD-, Procedure- and ATC-codes*

| **Category** | **Type** | **Codes** |
| --- | --- | --- |
| **Study population** |  |  |
| IE-diagnosis | ICD-10 | I33.x, I38.x, I39.8 |
|  | ICD-8 | 421 |
| **Surgery and prosthetic heart valves** |  |  |
| Aortic valve  *Repair*  *Prosthesis*  Mechanical  Biological | Danish procedure codes for medical examinations | KFMA20, KFMA32, KFMA96, KFMB00, KFMB10, KFMB20, KFMB96, KFMC00, KFMC00, KFMC10, KFMC20, KFMC96, KFMW96  KFMD, KFCA60, KFCA70  KFMD00, KFCA60  KFMD10, KFMD11, KFMD14, KFMD20, KFMD30, KFMD33, KFMD40, KFMD96, KFCA70 |
| Mitral valve  *Repair*  *Prosthesis*  Mechanical  Biological | Danish procedure codes for medical examinations | KFKA96, KFKB00, KFKB10, KFKB96, KFKC00, KFKC10, KFKC20, KFKC40, KFKC50, KFKC60, KFKC96, KFKW96  KFKD  KFKD00  KFKD10 |
| Pulmonary valve  *Repair*  *Prosthesis* | Danish procedure codes for medical examinations | KFJ  KFJB00, KJFB10, KFJB96, KFJC00, KFJC10, KFJC12, KFJD10, KFJD96, KFJE42  KFJF.x^1^ |
| Tricuspid valve  *Repair*  *Prosthesis* | Danish procedure codes for medical examinations | KFG  KFGC00, KFGC10, KFGC96, KFGD00, KFGD03, KFGD10, KFGD30, KFGD40, KFGD96,  KFGE.x^1^ |
| **Comorbidities** |  |  |
| Cardiac implantable electronic devices | Danish procedure codes for medical examinations | BFCA0, BFCB0 |
| Extraction of cardiac implantable electronic devices | Danish procedure codes for medical examinations | BFCA4, BFCB4, BFCB8 |
| Diabetes | ICD-10 | E10.x to E14.x |
|  | ICD-8 | 250 |
| Dialysis (chronic) | Danish procedure codes for medical examinations | BJFD2 |
| Renal disease | ICD-10 | E10.2, E11.2, E13.2, E14.2  I12.0 I13.x  N02.x to N08.x, N11.x, N12.x, N14.x, N15.8, N15.9, N16.0, N16.2 to N16.4, N16.8, N18.x, N19.x, N26.x  Q61.2, Q61.3, Q61.5, Q61.9  Z99.2 |
|  | ICD-8 | 25002  403, 404, 40039  581, 582, 583, 584, 59009, 59320  75310, 75311, 75319 |
| Liver disease | ICD-10 | B15.x to B19.x, C22.x, D68.4C I98.2, K70.x to K77.0, Q61.8A, Z94.4 |
|  | ICD-8 | 070, 155, 571 to 573 |
| Atrial fibrillation/flutter | ICD-10 | I48 |
|  | ICD-8 | 42793, 42794 |
| Congestive heart failure | ICD-10 | I11.0, I13.0, I13.2, I42.x, I50.x  J81.9 |
|  | ICD-8 | 425, 428 |
| Myocardial infarction | ICD-10 | I21.x, I22.x |
|  | ICD-8 | 410 to 414 |
| Disease of the mitral valve | ICD-10 | I34 |
|  | ICD-8 | 394, 396 |
| Disease of the aortic valve | ICD-10 | I35 |
|  | ICD-8 | 395, 396 |
| Ischemic/haemorrhagic stroke | ICD-10 | I60.x to I64.x |
|  | ICD-8 | 430 to 436 |
| Chronic obstructive lung disease | ICD-10 | J42, J43, J44 |
|  | ICD-8 | 490, 491, 492 |
| Malignancy | ICD-10 | C00 to C97 |
|  | ICD-8 | 140 to 209 |
| Drug abuse | ICD-10 | F11.2, F13.2, F14.2, X62.x |
| **Medicine** |  |  |
| Antihypertensive medication | ATC | C02A, C02B, C02C, C02DA, C02DB, C02DD, C02DG, C02L, C03A-B, C03D-E, C03X, C07A-D, C07F, C08 C08G, C09AA, C09BA, C09BB, C09CA, C09DA, C09DB, C09XA02, C09XA52 |
| Lipid Lowering medication | ATC | C10 |
| Oral anticoagulants  *Vitamin-K antagonists*  *NOAC^2^* | ATC | B01AA  B01AF01, B01AF02, B01AF03, B01AE07 |
| Glucose Lowering medication | ATC | A10A, A10B |
| “.x” indicates every sub-classification of diagnosis code or ATC code  ^1^ No differentiation was made between type of prosthesis (i.e. mechanical or biological) for pulmonary- or tricuspid-valve replacements.  ^2^ New Oral Anticoagulant  IE: Infective endocarditis. ATC: Anatomical Therapeutic Chemical Classification System.  ICD: International Classification of Diseases. NOMESCO: Danish procedure codes for medical examination organized by The Nordic Medico-Statistical Committee. | | |

*Supplementary Table 1. The table shows specific codes used to derive comorbidities and concomitant pharmacotherapy as well as type of valve surgery both prior to and during admission.*

**Supplementary Table 2**

*Supplementary Table 2: Type of valve-procedure during admission.*

|  | **Overall**  **N=1,981 (100%)** | **1999-2003**  **N=360 (100%)** | **2004-2008**  **N=483 (100%)** | **2009-2013**  **N=553 (100%)** | **2014-2018**  **N=585 (100%)** |
| --- | --- | --- | --- | --- | --- |
| **Replacement^1^** | **1,788 (90.3%)** | **331 (91.9%)** | **428 (88.6%)** | **493 (89.2%)** | **536 (91.6%)** |
| Aortic valve (single)^2^ | 1,121 (62.7%) | 206 (62.2%) | 279 (65.2%) | 313 (63.5%) | 323 (60.3%) |
| *Biological* | *759 (67.7%)* | *99 (48.1%)* | *167 (59.9%)* | *229 (73.2%)* | *264 (81.7%)* |
| *Mechanical* | *362 (32.3%)* | *107 (51.2%)* | *112 (40.1%)* | *84 (26.8%)* | *59 (18.3%)* |
| Mitral valve (single)^2^ | 424 (23.7%) | 92 (27.8%) | 103 (24.1%) | 106 (21.5%) | 123 (22.9%) |
| *Biological* | *214 (50.5%)* | *28 (30.4%)* | *30 (29.1%)* | *71 (67.0%)* | *85 (69.1%)* |
| *Mechanical* | *210 (49.5%)* | *64 (69.6%)* | *73 (70.9%)* | *35 (33.0%)* | *38 (30.9%)* |
| Aortic+mitral valves | 206 (11.5%) | 25 (7.6%) | 43 (10.0%) | 63 12.8%) | 75 (14.0%) |
| Right-side valves | 22 (1.2%) | <4 (<1.2%) | <4 (<0.9%) | 8 (1.6%) | 10 (1.9%) |
| Left+right sided valves | 15 (0.8%) | 5 (1.5%) | <4 (<0.9%) | <4 (<0.8%) | 5 (0.9%) |
| **Repair only** | **193 (9.7%)** | **29 (8.1%)** | **55 (11.4%)** | **60 (10.8%)** | **49 (8.4%)** |
| Aortic valve (single) | 10 (5.2%) | <4 (<13.8%) | <4 (<7.3%) | <4 (<6.7%) | <4 (<8.2%) |
| Mitral valve (single) | 148 (76.7%) | 20 (69.0%) | 44 (80.0%) | 44 (73.3%) | 40 (81.6%) |
| Aortic+mitral valves | 6 (3.1%) | <4 (<13.8%) | <4 (<7.3%) | <4 (<6.7%) | <4 (<8.2%) |
| Right-side valves | 29 (15.0%) | 6 (20.7%) | 8 (14.5%) | 11 (18.3%) | 4 (8.2%) |
| ^1^ If patients had both a replacement- and repair-procedure during their admission, they were counted as having a replacement-procedure.  ^2^ If patients had both a mechanic and a biological valve during their admission, the type of the last valve was registered. | | | | | |

*Supplementary Table 2. The table shows the distribution of surgery-type pr. calendar period during admission.*

| **Supplementary Table 3**   \| *Supplementary Table 3: Odds ratio of valve surgery during admission for prespecified covariates in the study period 1999-2018 overall and when stratifying by calendar periods.* \| \| \| \| \| \| \| \| \| --- \| --- \| --- \| --- \| --- \| --- \| --- \| --- \| \|  \|  \| **Overall model^1^** \| **Calendar period**  **1999-2003^2^** \| **Calendar period**  **2004-2008^2^** \| **Calendar period**  **2009-2013^2^** \| **Calendar period**  **2014-2018^2^** \| **P-value^3^** \| \| **Calendar period** \| 1999-2003 \| 1.00 (ref.) \| -- \| -- \| -- \| -- \| -- \| \|  \| 2004-2008 \| 1.14 (0.96-1.34) \| -- \| -- \| -- \| -- \| -- \| \|  \| 2009-2013 \| 1.20 (1.02-1.41) \| -- \| -- \| -- \| -- \| -- \| \|  \| 2014-2018 \| 1.10 (0.93-1.29) \| -- \| -- \| -- \| -- \| -- \| \| **Age group** \| <40 years \| 1.00 (ref.) \| 1.00 (ref) \| 1.00 (ref.) \| 1.00 (ref) \| 1.00 (ref.) \| *0.303* \| \|  \| 40-49 years \| 1.42 (1.11-1.82) \| 1.38 (0.84-2.26) \| 1.64 (1.00-2.68) \| 1.59 (0.98-2.58) \| 1.07 (0.63-1.79) \| \|  \| 50-59 years \| 1.48 (1.19-1.84) \| 1.98 (1.27-3.06) \| 2.09 (1.34-3.27) \| 1.27 (0.82-1.97) \| 0.90 (0.57-1.40) \| \|  \| 60-69 years \| 1.14 (0.93-1.41) \| 1.32 (0.86-2.04) \| 1.57 (1.02-2.40) \| 1.03 (0.68-1.55) \| 0.79 (0.52-1.21) \| \|  \| 70-79 years \| 0.67 (0.54-0.83) \| 0.67 (0.43-1.05) \| 0.74 (0.47-1.16) \| 0.66 (0.43-1.02) \| 0.52 (0.34-0.79) \| \|  \| ≥79 years \| 0.11 (0.08-0.14) \| 0.13 (0.06-0.27) \| 0.11 (0.06-0.21) \| 0.11 (0.06-0.20) \| 0.07 (0.04-0.12) \| \| **Sex** \| \| 1.38 (1.23-1.56) \| 1.30 (0.99-1.69) \| 1.41 (1.10-1.81) \| 1.57 (1.23-2.00) \| 1.29 (1.04-1.60) \| *0.656* \| \| **Prior prosthetic heart valve** \| \| 0.70 (0.58-0.85) \| 0.97 (0.56-1.69) \| 0.41 (0.26-0.65) \| 0.69 (0.47-0.99) \| 0.79 (0.57-1.10) \| *0.265* \| \| **Renal disease** \| \| 0.57 (0.47-0.70) \| 0.84 (0.49-1.46) \| 0.64 (0.42-0.99) \| 0.55 (0.39-0.78) \| 0.49 (0.35-0.68) \| *0.380* \| \| **Liver disease** \| \| 0.43 (0.34-0.54) \| 0.66 (0.39-1.12) \| 0.53 (0.33-0.84) \| 0.29 (0.18-0.46) \| 0.41 (0.27-0.61) \| *0.144* \| \| **Congestive heart failure** \| \| 0.64 (0.54-0.75) \| 0.68 (0.44-1.06) \| 0.81 (0.57-1.15) \| 0.66 (0.49-0.90) \| 0.49 (0.36-0.66) \| *0.123* \| \| **Ischemic/haemorrhagic stroke** \| \| 0.69 (0.57-0.84) \| 0.45 (0.24-0.83) \| 0.75 (0.49-1.14) \| 0.71 (0.49-1.01) \| 0.73 (0.52-1.01) \| *0.582* \| \| *Odds Ratio >1 = increased likelihood. Hazard ratio >1 = increased rate. Reference (ref.)*  ^1^ Model adjusted for: calendar period, age groups, prior prsothetic heart valve, diabetes, renal disease, liver disease, atrial fibrillation/flutter, vongestive heart failure, myocardial infarction, disease of the mitral valve, disease of the aortic valve, ischemic/haemorrhagic stroke, chronic obstructive lung disease, malignancy.  ^2^ Overall dataset was split by calendar periods and models were adjusted for same covariates as in ”^1^”.  ^3^ Analyses were performed on the overall dataset. P-values were reported for the test for interaction between each of the prespecified covariates and calendar period for valve surgery during admission. \| \| \| \| \| \| \| \| |
| --- | --- | --- | --- | --- | --- | --- | --- | --- | --- | --- | --- | --- | --- | --- | --- | --- | --- | --- | --- | --- | --- | --- | --- | --- | --- | --- | --- | --- | --- | --- | --- | --- | --- | --- | --- | --- | --- | --- | --- | --- | --- | --- | --- | --- | --- | --- | --- | --- | --- | --- | --- | --- | --- | --- | --- | --- | --- | --- | --- | --- | --- | --- | --- | --- | --- | --- | --- | --- | --- | --- | --- | --- | --- | --- | --- | --- | --- | --- | --- | --- | --- | --- | --- | --- | --- | --- | --- | --- | --- | --- | --- | --- | --- | --- | --- | --- | --- | --- | --- | --- | --- | --- | --- | --- | --- | --- | --- | --- | --- | --- | --- | --- | --- | --- | --- | --- | --- | --- | --- | --- | --- | --- | --- | --- | --- | --- | --- | --- | --- | --- | --- | --- | --- | --- | --- | --- | --- | --- | --- | --- | --- | --- | --- | --- | --- | --- | --- |

*Supplementary Table 3. The table shows the odds ratio (OR) of surgery during admission for prespecified covariates in the study period 1999-2018. Also, estimates are shown when stratifying the data by calendar period (1999-2003, 2004-2008, 2009-2013, 2014-2018). The P-value for interaction is derived from an analysis of each covariates’ effect modification on calendar periods’ effect on surgery during admission.*

**Supplementary Table 4**

| *Supplementary Table 4: Odds ratio of valve surgery during admission and hazard ratio of 30-day postoperative mortality for patients with infective endocarditis in the study period 2000-2018 including CIED and dialysis.* | | | | | |
| --- | --- | --- | --- | --- | --- |
|  | | **Valve surgery during admission** |  | **30-day postoperative mortality** |  |
| *Calendar periods* | | *Odds ratio (95% CI)*  *Adjusted^1^* | *P-value* | *Hazard ratio (95% CI)*  *Adjusted^2^* | *P-value* |
| 2000-2003 |  | 1.00 (ref.) | -- | 1.00 (ref.) | -- |
| 2004-2008 |  | 1.18 (0.99-1.42) | *0.068* | 0.99 (0.65-1.51) | *0.973* |
| 2009-2013 |  | 1.30 (1.09-1.55) | *0.004* | 0.44 (0.28-0.70) | *<0.001* |
| 2014-2018 |  | 1.20 (1.01-1.43) | *0.042* | 0.56 (0.37-0.87) | *0.010* |
| *Odds Ratio >1 = increased likelihood. Hazard ratio >1 = increased rate. Reference (ref.)*  *^1^Model adjusted for: calendar period, age groups, sex, prior prosthetic heart valve, diabetes, renal disease, liver disease, atrial fibrillation/flutter, congestive heart failure, myocardial infarction, disease of the mitral valve, disease of the aortic valve, ischemic/haemorrhagic stroke, chronic obstructive lung disease, malignancy. ^2^Model adjusted for: calendar period, age (continues), sex, prior prosthetic heart valve, diabetes, renal disease, liver disease, atrial fibrillation/flutter, congestive heart failure, myocardial infarction, disease of the mitral valve, disease of the aortic valve, ischemic/haemorrhagic stroke, chronic obstructive lung disease, malignancy.* | | | | | |

*Supplementary Table 4. The table shows the associated odds ratios of valve surgery and the hazard ratio of 30-day postoperative mortality when adjusting for covariates (including: CIED and dialysis) for patients with first-time infective endocarditis between 2000-2018.*
